# Psychiatric risk gene *Cacna1c* determines mitochondrial resilience against oxidative stress in neurons

**DOI:** 10.1038/s41419-018-0676-9

**Published:** 2018-05-29

**Authors:** Susanne Michels, Markus Wöhr, Rainer KW Schwarting, Carsten Culmsee

**Affiliations:** 1 Institute of Pharmacology and Clinical Pharmacy, University of Marburg, Marburg, Germany; 2 Department of Experimental and Biological Psychology, University of Marburg, Marburg, Germany; 3 Center for Mind, Brain and Behavior, University of Marburg, Marburg, Germany

Neuropsychiatric disorders, including major depression (MDD) and bipolar disorder (BD) are highly heritable and their etiologies involve complex interactions between genetic and environmental risk factors^1^. *CACNA1C*, which codes for the α_1C_ subunit of the l-type calcium channel (LTCC) Ca_V_1.2, has been identified by several genome-wide association studies as one of the strongest and most replicable genetic risk factors for affective disorders such as MDD and BD^2^. In the brain, Ca_V_1.2 plays a pivotal role in modulating gene transcription, synaptic plasticity, and cell survival^3^. However, the underlying mechanisms explaining how genetic alterations in *CACNA1C* affect the risk for neuropsychiatric disorders remain largely unknown.

Besides genetic predispositions, various environmental influences (comprising adverse life events such as childhood maltreatment, migration, or chronic stress) contribute to disease susceptibility^4^. As reported previously, impaired cellular adaptation to environmental stressors leads to the activation of oxidative stress pathways, thereby causing oxidative damage to membrane lipids, proteins, and in particular mitochondria^5^. Consequently, increasing evidence suggests a crucial role for mitochondrial dysfunction and related key determinants of cellular stress, e.g., impaired calcium homeostasis and excessive reactive oxygen species (ROS) formation, in the development of major neuropsychiatric disorders^6^. Furthermore, mitochondrial dysfunction is currently being discussed as a potential biomarker for affective disorders, supporting early diagnosis, control of disease progression, and evaluation of treatment response^7^.

Our recent findings published in *Cell Death Discovery* provide novel insight into a gene × stress interaction by showing that reduced *Cacna1c* expression mediated neuroprotective effects against oxidative stress, predominantly at the level of mitochondria^8^. In this study, we used immortalized mouse hippocampal HT22 cells, a well-established model system to investigate glutamate-induced oxidative stress, which reflects a common cellular response to environmental stress^9^. As summarized in Fig. 1, we could demonstrate that both siRNA-mediated *Cacna1c* gene silencing and LTCC blockade with the dihydropyridine (DHP) nimodipine significantly prevented the glutamate-induced rise in lipid peroxidation, excessive ROS formation, collapse of mitochondrial membrane potential, loss of ATP, reduction in mitochondrial respiration, and ultimately oxidative cell death. In addition, downregulation of *Cacna1c* substantially diminished the elevation in mitochondrial calcium levels 16 h after glutamate treatment. This effect is likely attributed to reduced calcium influx through plasma membrane-localized Ca_V_1.2 channels. Moreover, both *Cacna1c* knockdown and pharmacological LTCC inhibition led to altered Ca_V_1.2-dependent gene transcription regulation, thereby suppressing the enhanced expression of the inner mitochondrial membrane calcium uptake protein MCU upon glutamate exposure^8^. In the employed paradigm of oxidative glutamate toxicity, *Cacna1c* depletion also protected against detrimental mitochondrial fission and stimulated mitochondrial biogenesis without affecting mitophagy, thus promoting the turnover of mitochondria and preventing the accumulation of dysfunctional mitochondria in neuronal HT22 cells. Collectively, these data imply that upstream genetic modifications, e.g., reduced *CACNA1C* expression, converge to control mitochondrial function, resulting in cellular resilience against oxidative stress^6^.

**Fig. 1:**
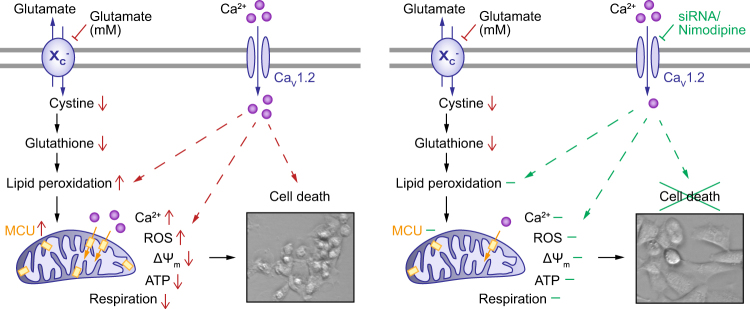
*Cacna1c* downregulation preserved mitochondrial function in glutamate-challenged neuronal HT22 cells. As mouse hippocampal HT22 cells do not express functional ionotropic glutamate receptors, glutamate toxicity is mediated via an oxidative stress-dependent pathway, including inhibition of the glutamate/cystine antiporter, a subsequent depletion of glutathione and a consecutive impairment of mitochondrial function, which ultimately leads to neuronal cell death (left panel). This glutamate-induced cascade is positively affected by *Cacna1c* knockdown (siRNA) and pharmacological LTCC inhibition (Nimodipine), which both mediate substantial protective effects on lipid peroxidation, mitochondrial integrity and function, and cell viability (right panel). X_C_^−^, glutamate/cystine antiporter; Ca_V_1.2, voltage-gated l-type calcium channel; MCU, mitochondrial calcium uniporter; ROS, reactive oxygen species; ΔΨ_m_, mitochondrial membrane potential; ATP, adenosine triphosphate

So far, both decreased and increased Ca_V_1.2 levels have been associated with the main non-coding risk single-nucleotide polymorphism (SNP) rs1006737, suggesting that alternations in *CACNA1C* expression may be developmental-stage-, brain-region-, as well as cell-type-specific^10, 11^. In this context, it has been shown that *Cacna1c* depletion in forebrain glutamatergic neurons, either during development or adulthood, differentially modulates synaptic plasticity, stress susceptibility, and cognition in mice^12^. These findings indicate an essential role for Ca_V_1.2 in memory formation during development, whereas Ca_V_1.2 activation during adulthood is even detrimental for synaptic plasticity. Accordingly, using a newly developed heterozygous *Cacna1c* rat model, Kisko et al.^13^ recently found that *Cacna1c* haploinsufficiency led to pro-social 50-kHz ultrasonic communication deficits during the critical developmental period of adolescence. On the contrary, in adult mice, both heterozygous *Cacna1c* knockout and DHP LTCC blockade are associated with antidepressant-like behavior and resilience to chronic stress^14^; beneficial phenotypes, which are more in line with the neuroprotective effects that we observed in conditions of reduced *Cacna1c* expression combined with oxidative stress.

Overall, the current controversy regarding the direction and effects of an altered *CACNA1C* expression emphasizes the complex and heterogeneous nature of affective disorders, which cannot be characterized by a single pathway. In this regard, we are fully aware that on the basis of the applied cellular model system, clinical and therapeutic implications from our findings are limited. However, accumulating evidence suggests that mitochondrial dysfunction contributes to disease neuropathology and may therefore represent a converging point of alterations in complex interdependent processes involved in energy metabolism and calcium homeostasis^15^. Thus, by establishing a link between *Cacna1c* and mitochondria in the context of oxidative stress, our study adds to a better understanding of the intracellular processes likely involved in the pathophysiology of *CACNA1C*-associated disorders.

## References

[1] Keers R, Uher R (2012). Gene-environment interaction in major depression and antidepressant treatment response. Curr. Psychiatry Rep..

[2] Group CrossDisorderofthePsychiatricGenomicsConsortium (2013). Identification of risk loci with shared effects on five major psychiatric disorders: a genome-wide analysis. Lancet.

[3] Bhat S (2012). CACNA1C (Cav1.2) in the pathophysiology of psychiatric disease. Prog. Neurobiol..

[4] Pignon B (2017). Prevalence and clinical severity of mood disorders among first-, second- and third-generation migrants. J. Affect Disord..

[5] Salim S (2017). Oxidative stress and the central nervous system. J. Pharmacol. Exp. Ther..

[6] Manji H (2012). Impaired mitochondrial function in psychiatric disorders. Nat. Rev. Neurosci..

[7] Sigitova E, Fišar Z, Hroudová J, Cikánková T, Raboch J (2017). Biological hypotheses and biomarkers of bipolar disorder. Psychiatry Clin. Neurosci..

[8] Michels S (2018). Downregulation of the psychiatric susceptibility gene Cacna1c promotes mitochondrial resilience to oxidative stress in neuronal cells. Cell Death Discov..

[9] Tobaben S (2011). Bid-mediated mitochondrial damage is a key mechanism in glutamate-induced oxidative stress and AIF-dependent cell death in immortalized HT-22 hippocampal neurons. Cell Death Differ..

[10] Gershon ES (2014). A rare mutation of CACNA1C in a patient with bipolar disorder, and decreased gene expression associated with a bipolar-associated common SNP of CACNA1C in brain. Mol. Psychiatry.

[11] Yoshimizu T (2015). Functional implications of a psychiatric risk variant within CACNA1C in induced human neurons. Mol. Psychiatry.

[12] Dedic N (2018). Cross-disorder risk gene CACNA1C differentially modulates susceptibility to psychiatric disorders during development and adulthood. Mol. Psychiatry.

[13] Kisko T. M. et al. Cacna1c haploinsufficiency leads to pro-social 50-kHz ultrasonic communication deficits in rats. *Dis. Model. Mech*. (2018). 10.1242/dmm.034116. Epub ahead of print. Published 8 May 2018. 10.1242/dmm.034116 PMC6031367 29739816

[14] Kabir ZD, Martínez-Rivera A, Rajadhyaksha AM (2017). From gene to behavior: L-type calcium channel mechanisms underlying neuropsychiatric symptoms. Neurotherapeutics.

[15] Machado AK, Pan AY, da Silva TM, Duong A, Andreazza AC (2016). Upstream pathways controlling mitochondrial function in major psychosis. a focus on bipolar disorder. Can. J. Psychiatry.

